# Study the removal of copper ions from wastewater using an array of horizontal rough vibrating zinc discs

**DOI:** 10.1038/s41598-026-52620-6

**Published:** 2026-05-20

**Authors:** S. E. Tafeh, S. A. Nosier, G. H. Sedahmed, D. A. Elgayar

**Affiliations:** Chemical Engineering Department, Faculty of Engineering, Alexandria University, Alexandria, Egypt

**Keywords:** Cementation, Wastewater treatment, Copper recovery, Mass transfer, Electro-winning of zinc, Surface roughness, Engineering, Chemical engineering

## Abstract

The removal of copper ions from synthetic wastewater was investigated using cementation on arrays of oscillating smooth and rough (corrugated) zinc discs under different conditions initial copper ions concentration, vibration intensity (frequency, amplitude), spacing between zinc discs, surface roughness (peak to valley height) and temperature. The results showed that the mass transfer coefficient increased significantly with increasing vibration intensity, initial copper ion concentration, disc spacing, degree of surface roughness, and temperature. Rough (corrugated) discs exhibited significantly higher mass transfer rates than smooth discs, emphasizing the critical role of surface roughness in enhancing diffusion-controlled cementation. The effect of temperature was found to fit Arrhenius equation with an activation energy of 2.58 $\mathrm{k}\mathrm{c}\mathrm{a}\mathrm{l}/\mathrm{m}\mathrm{o}\mathrm{l}\mathrm{e}$ which confirms the diffusion-controlled nature of the reaction. The cementation rates were expressed in terms of the mass transfer coefficient. Dimensional analysis was performed to correlate the mass transfer data, resulting in two separate correlations for the smooth and corrugated arrays. These correlations provide a reliable basis for the design and scale-up of vibratory cementation reactors from bench to industrial scale. The present data fit the correlations Sh = 0.0134 Re^1.06^ Sc^0.33^ (S/d_c_)^0.46^ for the array of smooth zinc discs, Sh = 0.0711 Re^0.96^ Sc^0.33^ (S/d_c_)^−0.49^ (P/d_c_)^0.49^ for array of rough zinc discs under the conditions: 3926.54 < Re < 70,115.28, 1397.2 < Sc < 1538.2, 0.14 < S/d_c_ < 0.36, and 0.014 < P/ d_c_ < 0.036 for rough discs.

## Introduction

Industrial wastewater treatment has become increasingly essential due to the growing discharge of toxic and non-biodegradable heavy metals into aquatic systems. Ions such as Cu^2^⁺, Hg^2^⁺, Cr⁶⁺, and Cd^2^⁺ pose severe environmental and health hazards because they accumulate in water, soil, and living organisms^1^. Consequently, developing efficient and economically viable methods to remove such contaminants remains a major focus in environmental and hydrometallurgical research. Copper is of particular concern due to its extensive industrial use in electroplating, electroless copper deposition, metal finishing, acid pickling, mining operations, and printed-circuit manufacturing^1^. Significant copper ions contamination also originates from domestic plumbing systems^2^. Recovering copper ions from industrial effluents is therefore important both for minimizing environmental damage and for reclaiming valuable metal resources. Among the available treatment methods- ion exchange^3^, adsorption^4^, electrochemical reduction^5^, precipitation^6^, reverse osmosis^7^ and cementation stands out for its simplicity, low cost, and dual benefit of metal removal and recovery^8^. Cementation of copper ions on zinc metal is known to be diffusion-controlled; therefore, hydrodynamic enhancement is critical for improving overall reactor performance^8,11^. A variety of configurations has been proposed to enhance mass transfer during cementation, including reciprocating perforated discs (Mubarak et al., El-Shazly et al.)^12,13^, rotating discs^14^ and cylinders^15^, rotating packed beds^16^, ultrasonic vibration^17^, gas-sparged systems^18^, modified stirred tank reactors^19^, jet reactors^20^, bubble column reactors^21^, fixed beds^22–24^, pulsating fixed beds^25^, and fluidized bed at stationary surface^26^. However, most previous studies on vibratory-disc systems have focused on perforated discs, which generate hydrodynamic patterns^12,13^ that differ significantly from those produced by unperforated horizontal discs. To date, the specific influence of disc surface topography- particularly engineered corrugation has not been systematically investigated in oscillating-disc cementation systems, despite the well-established role of surface roughness in promoting micro-turbulence and increasing the effective surface area available for diffusion-controlled reactions. This gap in the literature limits the ability of existing models to accurately predict cementation behavior in reactors incorporating structured surfaces. Furthermore, horizontal unperforated discs offer several potential advantages that have not been examined in earlier vibrating-disc studies: (i) a larger solid–liquid interfacial area compared to perforated plates, (ii) distinct oscillatory boundary-layer development, and (iii) potentially improved energy efficiency relative to rotary reactors at comparable mass transfer rates^27,28^.

To address these gaps, the present study investigates the cementation of copper ions on arrays of horizontal oscillating zinc discs with two surface configurations: smooth and corrugated. The use of corrugated discs with controlled geometric parameters (peak-to-valley height) enables, for the first time, a quantitative assessment of the contribution of surface roughness to mass transfer enhancement under oscillatory flow conditions. By systematically varying key operating parameters- including initial copper ion concentration, vibration intensity, spacing between zinc discs, surface roughness and, temperature-this study establishes a clear distinction from prior studies and reveals the unique combined hydrodynamic effects of oscillation and engineered surface structures. In addition, the study develops two new dimensionless design correlations—one for smooth discs and one for corrugated discs, providing a predictive framework for scaling up oscillating-disc cementation reactors from laboratory to industrial scale. These contributions fill a significant gap in the literature, and offer new insights into the performance of horizontal rough oscillating discs compared to previously, which has not been previously addressed.

## Experimental technique

### Apparatus

**Fig. 1:**
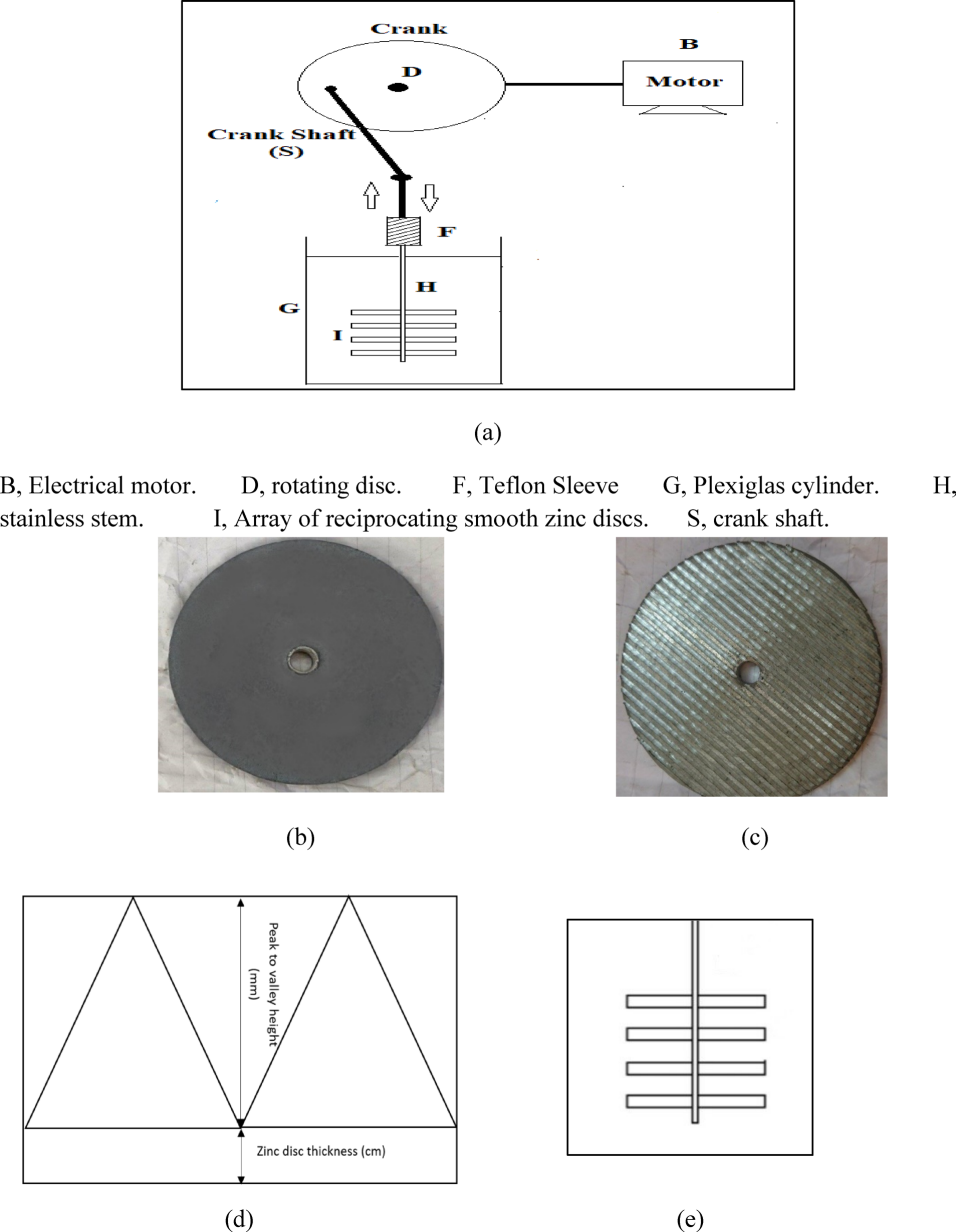
(**a**) Experimental Setup (**b**) Smooth Zinc Disc. (**c**) Corrugated Zinc Disc. (**d**) A schematic diagram of the rough disc surface from a side view. (**e**) A schematic diagram for the array of zinc discs.

Figure 1a shows the experimental setup used in the present study. It consists of a reactor and a mechanical vibrator system. The reactor is composed of a Plexiglas cylinder (G) of a diameter 15 cm and height 35 cm. An array of reciprocating zinc discs (I), which can be smooth or rough, as shown in Fig. 1b and c respectively, has a diameter of 7 cm and thickness of 0.8 cm; each array is composed of 4 discs. Roughness was introduced by cutting parallel grooves in the Zinc discs. The rough zinc discs had peak to valley heights ranged from 1 to 2.5 mm, which were measured using a digital micrometer to provide an accurate estimate of the surface roughness. Figure 1d illustrates a schematic of the rough disc surface from a side view. The spacing between discs ranged from 1 to 2.5 cm as shown in Fig. 1e. The array of smooth or rough zinc discs were placed in the center of the reactor at height of 5 cm from the bottom and distance of 4 cm from each side of the reactor. The array of reciprocating zinc discs was held inside the column by insulated stainless-stem (H) with a diameter of 3 mm, which passed through the centers of the discs. The disc array holder (H) was connected to a vibrator through a Teflon sleeve (F). The vibrator consists of a disc (D) rotated by a variable speed motor (B). The rotating motion of the disc was converted into reciprocating motion by means of a crank shaft (S) connected to the disc at a distance from its center. The amplitude was adjusted by changing the distance between the center of the rotating disc and the point of connection with the crank shaft. The frequency of oscillation was adjusted by controlling the motor speed of rotation by means of a veric and measured by an optical tachometer.

### Procedures

The rate of cementation of copper ions on an array of smooth zinc discs was determined by measuring the change in copper ions concentration over time. Prior to each run, the zinc discs were immersed in 10% dilute HCl to remove any oxide layer from the disc surface, then rinsed with distilled water and dried. A volume of 3.5L of freshly prepared copper sulphate solution was placed into the vessel, and the solution was simultaneously subjected to oscillatory motion at the required vibration intensity. All experiments lasted for 40 min. A 5 ml of sample was withdrawn every 5 min from solution using a calibrated pipette at different vibration intensities to monitor copper concentration during cementation process. The copper ions concentration was determined via iodometric titration^29^. Each 5 mL sample was transferred to a clean conical flask and acidified with 2–3 mL of 1 M H₂SO₄. Approximately 1 g of analytical-grade potassium iodide (KI, 99.5%) was added, causing copper ions to oxidize iodide ions and liberate iodine (I₂). The liberated iodine was titrated with a freshly prepared and standardized sodium thiosulfate (Na₂S₂O₃) solution, using starch as an endpoint indicator. The disappearance of the blue color marked the completion of the reaction. The volume of Na₂S₂O₃ consumed was used to calculate copper ions concentration based on the stoichiometry of the redox reaction^29^. All titrations were performed in triplicate to ensure precision and reproducibility. All chemical solutions were freshly prepared prior to each run.

All experiments were carried out at temperature 25 ± 2°C expect for those specifically performed to investigate the effect of temperature using a thermostatic bath, and, pH of all experiments was fixed at 5.4 ± 0.1 by continuous monitoring and adjustment with dilute solutions of H_2_SO_4_ or NaOH to prevent copper hydroxide precipitation. Solution viscosity and density, required for data correlation, were measured using an Ostwald viscometer and density bottle respectively^30^. Copper sulphate solution diffusivity was obtained from literature^31^. Measurement uncertainties were considered for key experimental devices: the pH meter (± 0.02 pH units), the thermometer (± 0.5 °C), and the optical tachometer for vibration frequency (± 0.1 Hz). These uncertainties, reflecting typical laboratory conditions using AR-grade chemicals, suggest a small propagated error in the calculated mass transfer coefficients, confirming the robustness of the reported data. A summary of the investigated parameters and their ranges is shown in Table 1.

**Table 1:** The investigated parameters and their ranges.

Studied parameter	Range
Initial copper concentration, M	0.01–0.03
Frequency, rpm	100–600
Amplitude, cm	0.5–1.5
Zinc discs type	Smooth or Rough
Peak to valley height, mm	1–2.5
Distance between discs, cm	1–2.5

## Results and discussion

The mass transfer coefficient which expresses the rate of cementation was calculated from the batch reactor design^32^.

1 $${\text{Q ln Co}}/{\mathrm{C}} = {\text{KA t}}$$

where Q is solution volume in the reactor; cm^3^, Co, C are initial concentration and the concentration of CuSO_4_ respectively_;_ M, t is time of reaction; s, A is the active surface area of the array of zinc discs, cm^2^; K is the mass transfer coefficient, cm/s. The above equation was obtained by integrating the material balance equation of batch reactor.

2 $$- {\mathrm{QdC}}/{\mathrm{dt}} = {\mathrm{KAC}}$$

**Fig. 2:**
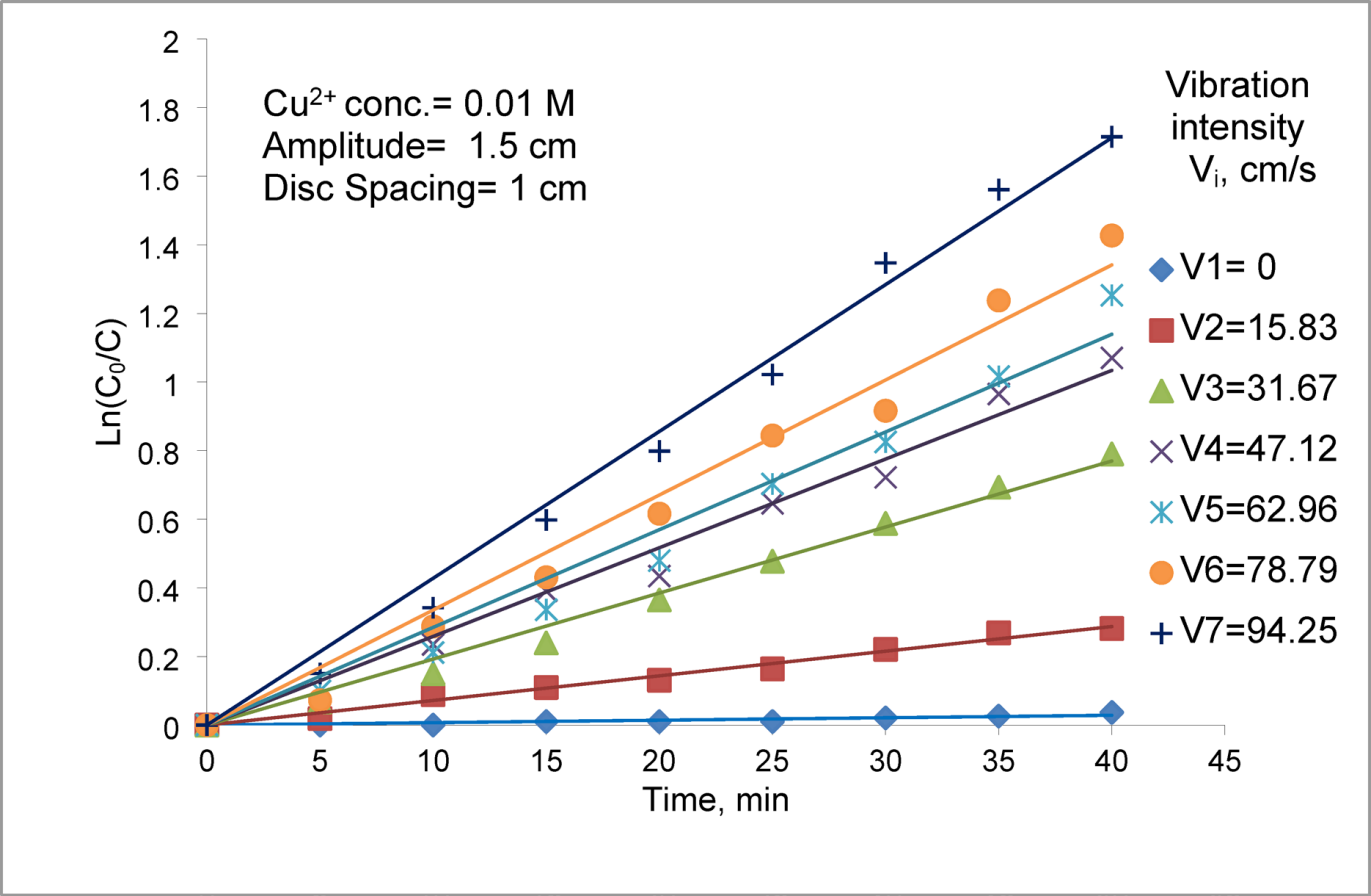
ln (C_o_/C) Vs. time at different vibrating intensities.

The mass transfer coefficient was obtained under different conditions by plotting ln Co/C versus time.The slope of the resulting straight line gives (KA/Q) from which K was calculated. Figure 2 shows plots of ln (C_o_/C) versus time at different vibration intensity. The present data confirm the first order kinetic model. Obtained by previous studies^9,22^ for cementation of copper on less noble metals.

### Effect of initial Copper ions concentration

**Fig. 3:**
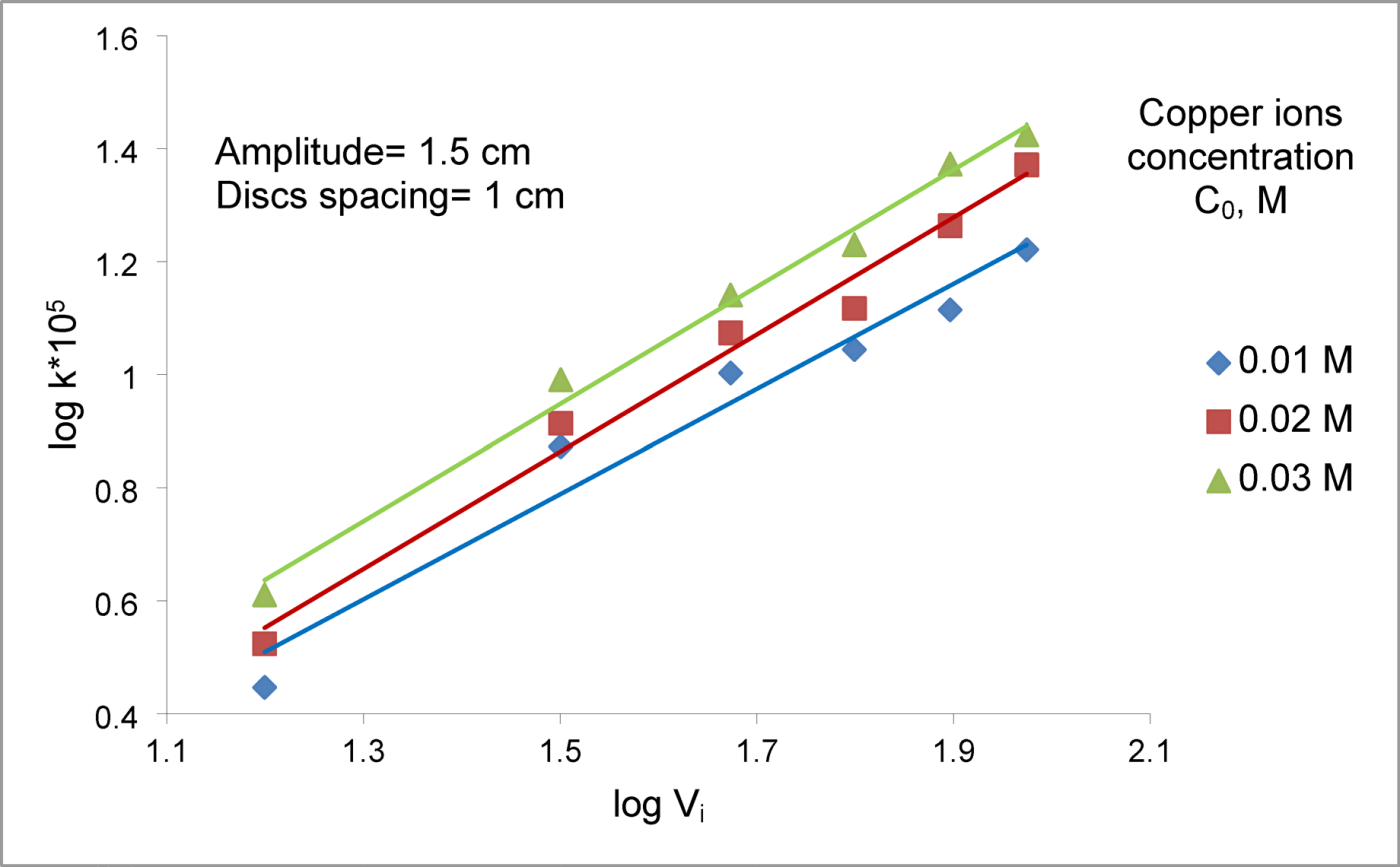
Effect of initial copper ions concentration on array of smooth zinc discs at different vibration intensity.

Figure 3 shows that the mass transfer coefficient increases with increasing initial copper ions concentration at different vibration velocities. This behavior is governed by two primary mechanisms. (I) The higher copper ions concentration in the bulk enhances the driving force for mass transfer, accelerating the cementation rate, as described by Eq. (2). This effect has been confirmed in recent studies on copper-zinc cementation, where elevated metal-ion concentrations increase interfacial mass transfer rates (Oliveira et al. 2019)^33^. (II) The increase in initial copper ions concentrations promote the formation of rough or dendritic copper deposits on the disc surface. This surface roughness induces local micro-turbulence and intensifies hydrodynamic mixing as the solution moves past the discs during pulsation, thereby enhancing both mass transfer and the rate of cementation^34^. Recent studies on mechanically assisted cementation confirm that such roughened copper surfaces significantly improve surface reactivity and interfacial hydrodynamics (Morsy et al., 2025, Scientific Reports)^35^. Conversely, higher initial copper sulfate concentration slightly reduces copper ions diffusivity due to stronger interionic interactions and increased solution viscosity^36^, which can decrease the mass transfer coefficient ($K=\frac{D}{\delta }$)^33^. Overall, the positive effects of increased driving force and micro-turbulence dominate over the diffusivity reduction, resulting in a net increase in the mass transfer coefficient with increasing initial copper ions concentration.

The quantity and composition of the cementation sludge were observed to be strongly dependent on the initial copper ions concentration in the solution. Higher copper ions concentrations increased the availability of ions for reduction and deposition onto the zinc discs, leading to a greater accumulation of metallic copper within the sludge. In addition, minor amounts of zinc oxides and hydroxides were detected, resulting from partial dissolution of the zinc substrate during the displacement reaction. The formation and chemical characteristics of the sludge are in agreement with the expected Zn–Cu displacement mechanism, confirming the system’s predictable behavior under varying copper ions concentrations. Recent studies on mechanically assisted cementation have similarly reported that both the initial metal-ion concentration and local hydrodynamic conditions significantly influence the quantity and morphology of the resulting metallic deposits^37^.

### Effect of frequency and amplitude (vibration intensity)

**Fig. 4:**
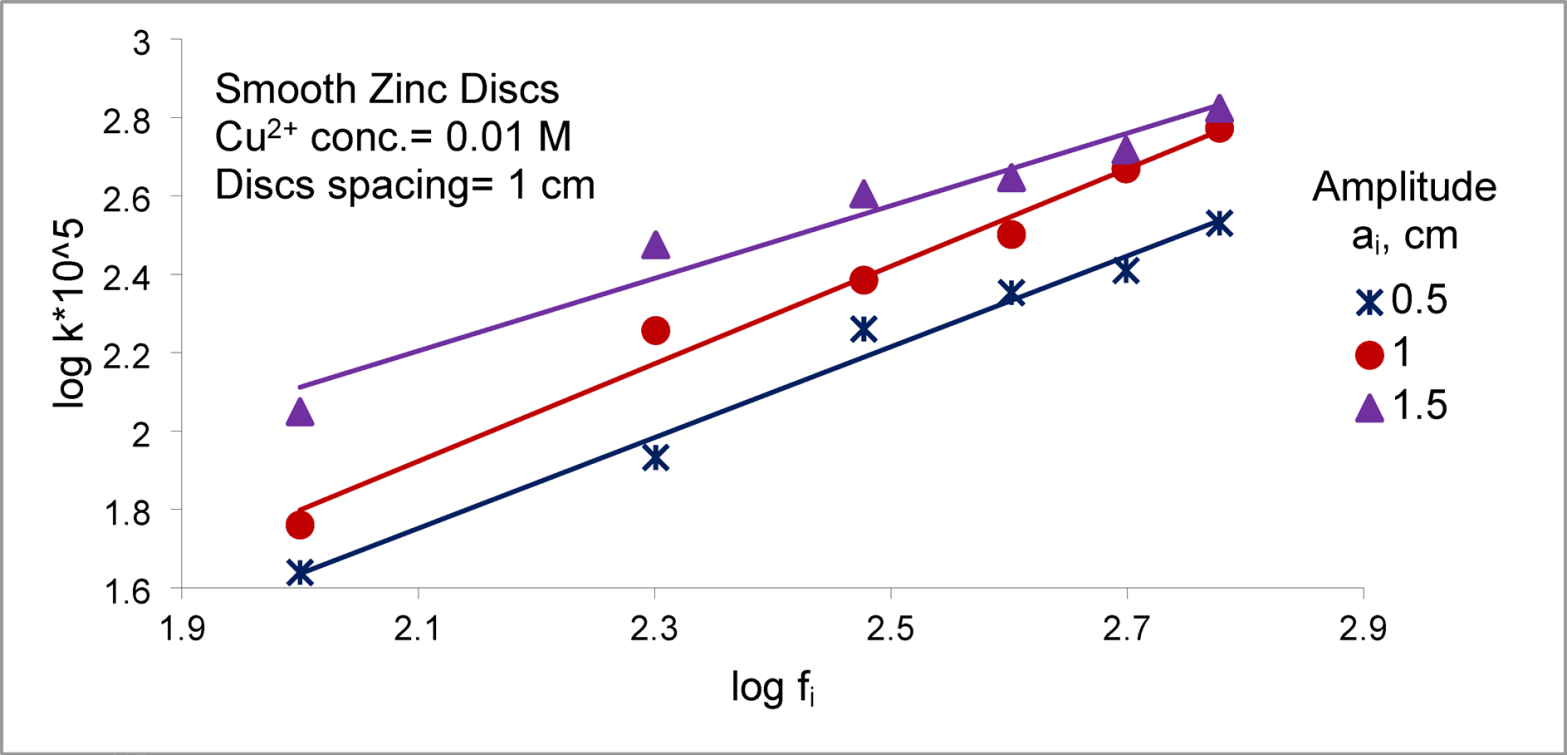
Effect of frequency of vibration on the mass transfer coefficient of copper cementation at different amplitude for array of smooth zinc discs.

**Fig. 5:**
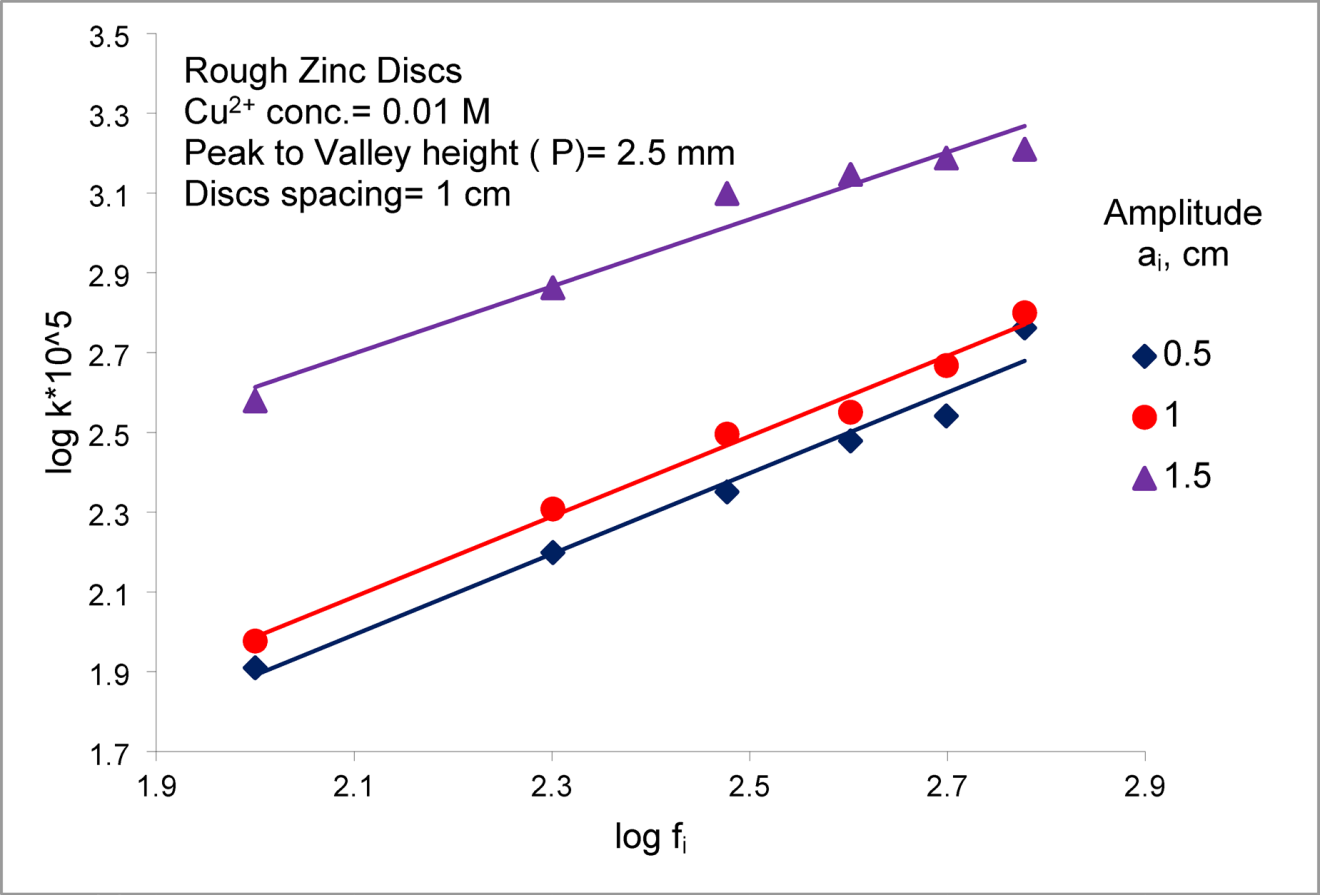
Effect of frequency of vibration on the mass transfer coefficient of copper cementation at different amplitude for array of rough zinc discs with peak to valley height 2.5 mm.

**Fig. 6:**
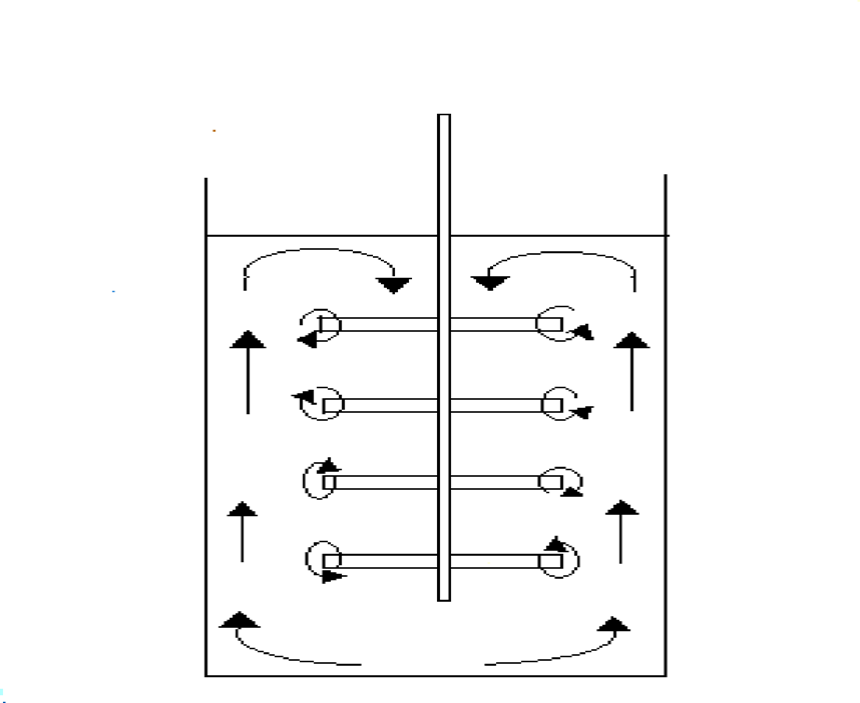
Approximate Flow Pattern during disc array vibration.

**Fig. 7:**
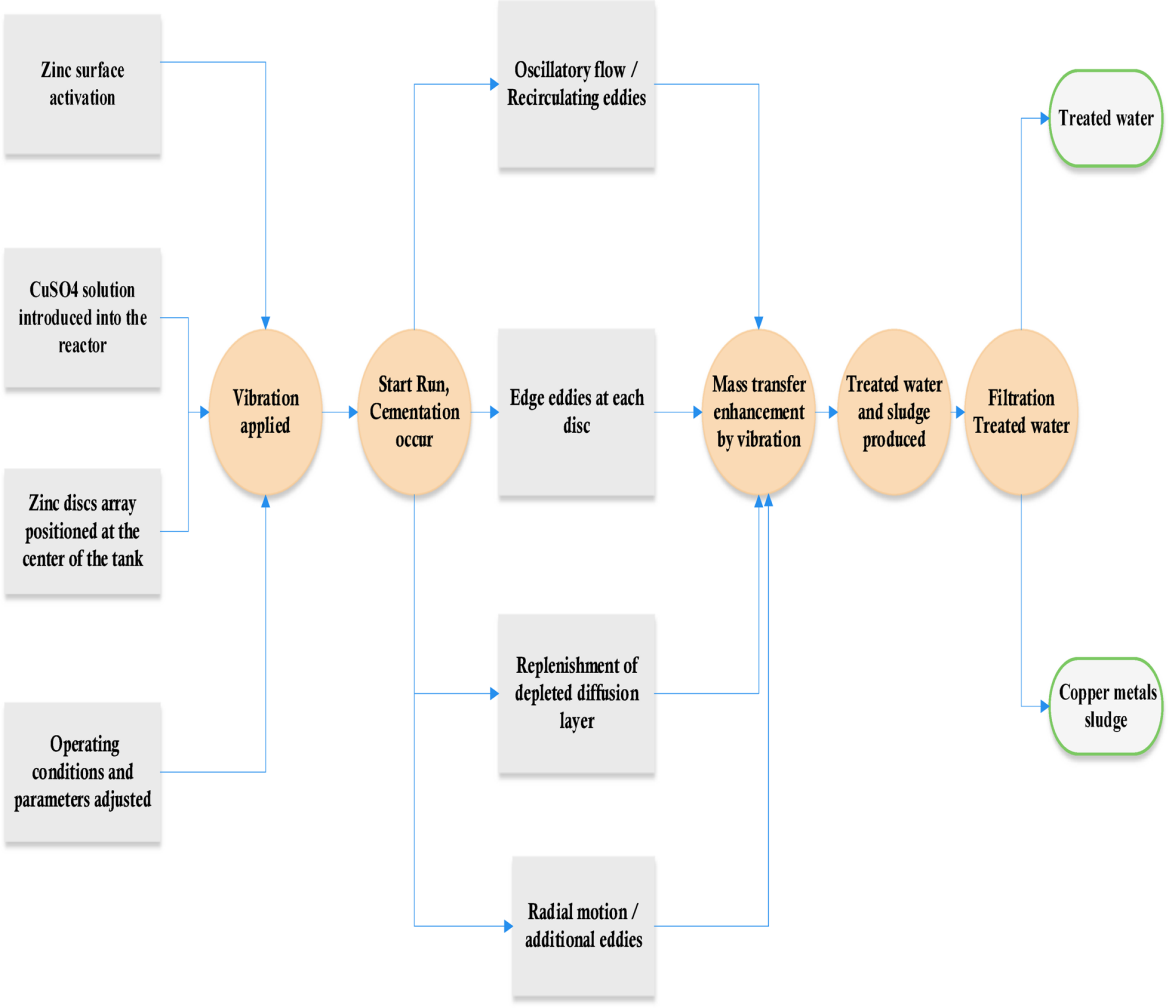
The vibration-assisted cementation mechanism.

Figures 4 and 5 illustrate that the mass transfer coefficient (K) increases with both frequency and amplitude (vibration intensity) V_i_ (V_i_ = a (2 π f_i_)) for an array of smooth zinc discs and an array of rough zinc discs with peak-to- valley 2.5 mm, respectively. Where a_i_ is amplitude, cm and f_i_ is frequency, RPS. Serval hydrodynamic mechanisms likely contribute synergistically to this enhancement:

I. the formation of oscillatory flow in the form of large recirculating eddies along the container wall height^38^ (Fig. 6). The induced steady secondary flow ( acoustic streaming) is caused by the interaction of viscous and inertial effects in the boundary layer of oscillating surface^38,39^ .The recirculating eddies flow (the oscillatory flow) reduce hydrodynamic boundary layer thickness and diffusion layer ( δ ) at the surface of smooth zinc discs (active area) with a consequent increase in the mass transfer coefficient (K = D/δ)^36^ and the rate of cementation especially at the upper and lower discs. Tojo et al.^40,44^ who studied the performance of vibration horizontal unperforated disc of smaller diameter than the container in suspending solid particles as a tracer, observed the presence of an upward circulating flow. The higher the vibration velocity, the higher the rate of upward flow circulation. This flow pattern supports the present data interpretation.
II. In addition to flow circulation induced by disc vibration, eddies are formed at the edge of each disc during vibration^45^ (Fig. 6). These eddies contribute to enhancing the rate of mass transfer and the rate of cementation at each disc especially at the peripheral disc area
III. As the disc moves up and down, the depleted solution in the diffusion layer at the disc surface is replenished with fresh bulk solution by diffusion from the copper ions- rich zones below and above the disc
IV. As the disc moves up and down, it induces radial motion of the solution past the disc surface with a consequent eddy generation which reduce the diffusion layer thickness^39^. Figure 7 summarizes the conceptual diagram illustrating the vibration-assisted cementation mechanism, showing the process from initial zinc surface activation to final copper deposition and treated water recovery.

**Fig. 8:**
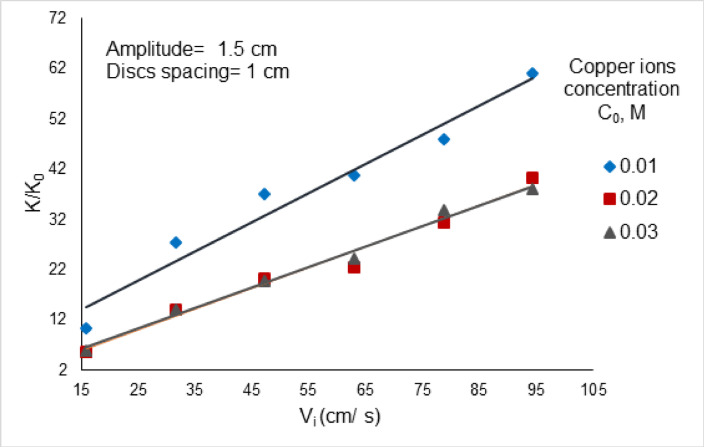
The enhancement ratio for vibrating array of smooth zinc discs for different concentration range from 0.01 to 0.03 M.

To appreciate the extent to which disc vibration enhances the rate of mass transfer and the rate of cementation, a series of experiments were conducted on cementation using stationary discs without vibration. Figure 8 shows the relation between the intensification factor (K_v_/K_0_) for various concentration range from 0.01 to 0.03 M versus V_i_, where K_v_ mass transfer coefficient by array of smooth zinc discs; cm/s, and K_0_ mass transfer coefficient of stationary non vibrating array of smooth zinc discs; cm/s. Table 2 shows the enhancement ratio for vibrating array of smooth zinc discs (K_v_/K_0_) for different concentration range from 0.01 to 0.03 M.

**Table 2:** The enhancement ratios at different vibration intensities for array of smooth zinc discs at different concentration from 0.01 to 0.03 M and different Reynold^’^s number, the value ranges from 5.73 to 61.14 depending on the operating conditions.

V_i_ (cm/s)	(K_v_/K_o_)_0.01M_	(K_v_/K_o_)_0.02M_	(K_v_/K_o_)_0.03M_
15.83	10.29	5.73	5.83
31.67	27.43	14.07	14
47.12	37	20.33	19.78
62.96	40.71	22.46	24.28
78.79	47.86	31.47	33.72
94.25	61.14	40.33	38

### Effect of array of zinc discs spacing

**Fig. 9:**
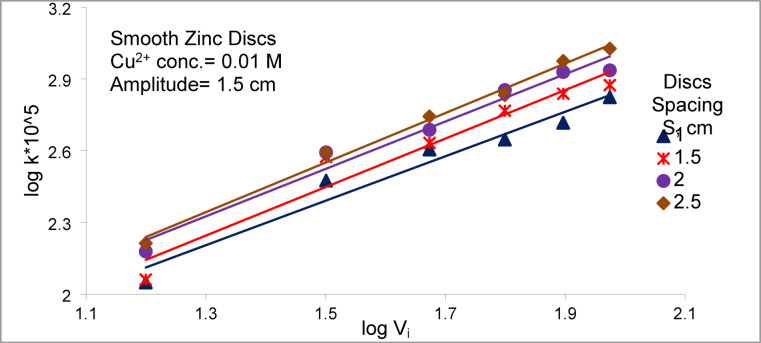
Effect of zinc discs spacing on array of smooth zinc discs at different vibration intensity.

Figure 9 shows that the mass transfer coefficient (K) increases significantly as the spacing between smooth zinc discs is increased from 1 to 2.5 cm under various vibration intensities. This trend can be rationalized through coupled hydrodynamic and diffusion-controlled mechanisms specific to zinc disc arrays. (I) First, as the inter-disc spacing increases, the solution volume between discs expands. Consequently, the deposition of copper does not lead to a substantial reduction in copper ions concentration within the local solution volume. Thus, the driving force (ΔC) for cementation remains largely maintained, in contrast to narrow gaps where rapid local depletion of copper ions concentration can significantly reduce ΔC. This phenomenon is consistent with observations in electrochemical and vibratory systems, where electrode spacing critically influences ion transport efficiency^46^. (II) Second, wider spacing facilitates enhanced fluid circulation around the zinc discs, reducing stagnant zones and allowing micro-eddies to form more freely. The oscillatory motion of the discs induces radial and rotational flows that disrupt the diffusion boundary layer ($\delta$ ) at the disc surfaces, leading to increased K values ($K=\frac{D}{\delta }$). The formation of such micro-eddies and improved lateral mixing has been demonstrated in recent studies on mechanically assisted mass transfer, confirming that intensified fluid dynamics near vibrating surfaces accelerates metal deposition^47^. (III) Third, the interaction of oscillatory flows with the geometric spacing of the discs promotes boundary-layer destabilization, which facilitates continuous replenishment of copper ions from the bulk solution. This mechanism is analogous to findings in high-performance flow reactors, where structured spacing induces additional eddies, disrupts local concentration gradients, and enhances overall mass transfer^48^. Together, these effects-maintained driving force, intensified fluid circulation, boundary-layer destabilization, and enhanced radial and rotational flows provide a mechanistic and coherent explanation for the observed increase in the mass transfer coefficient with increasing disc-array spacing. This emphasizes the critical role of geometric configuration in optimizing the cementation efficiency of copper ions on zinc discs under oscillatory conditions.

### Effect of peak to valley height (P)

**Fig. 10:**
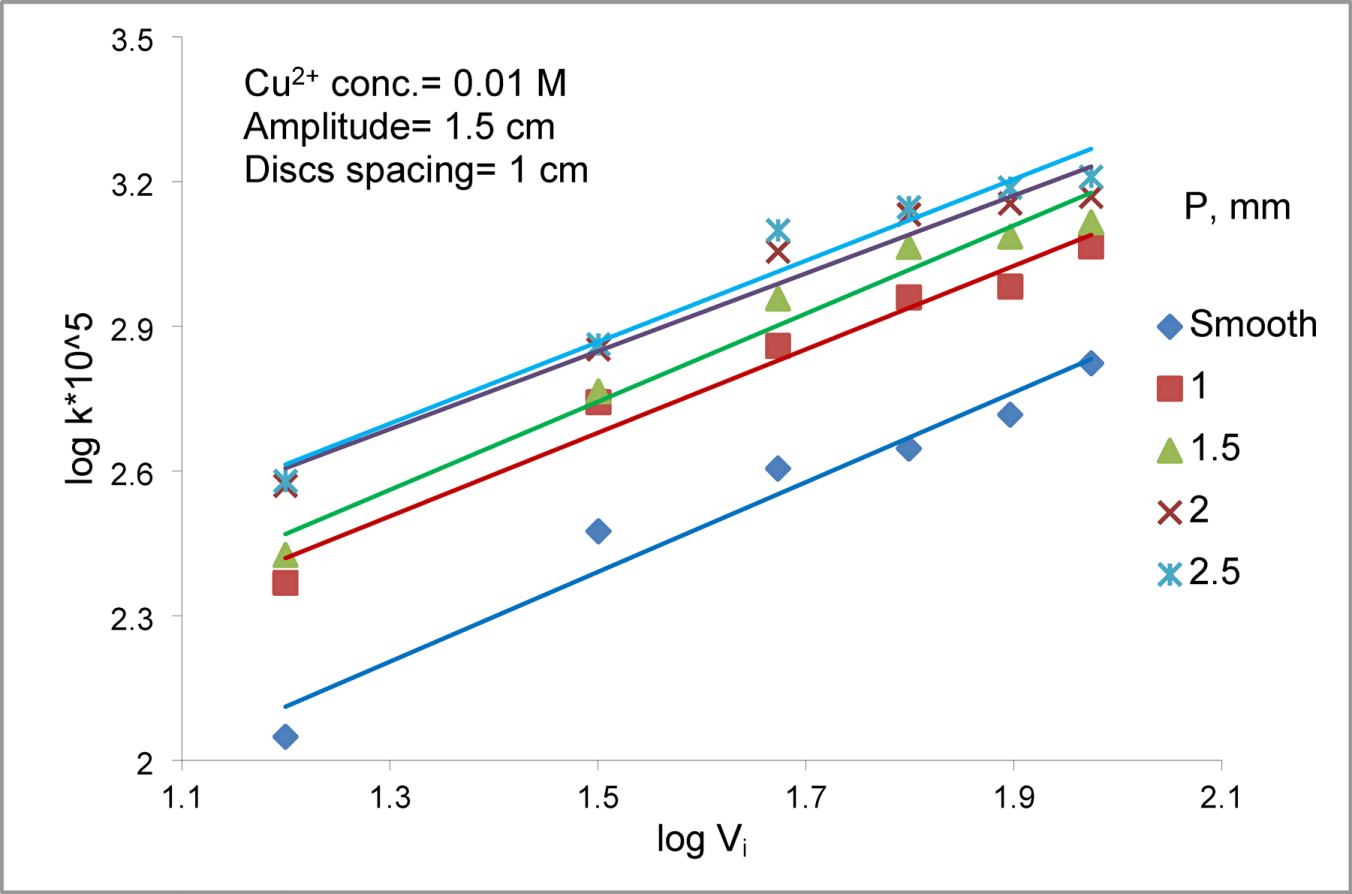
Effect of peak to valley height on array of rough zinc discs compared to array of smooth zinc discs at different vibration intensity.

Figure 10 shows the relation between log K Vs. log V_i_ for an array of smooth zinc discs compared with an array of rough zinc discs at different peak- to- valley height ranging from 1- 2.5 mm. The results indicate that oscillating rough discs consistently exhibit higher mass-transfer coefficients than smooth discs, with the enhancement factor ( ${K}_{r}/{K}_{s}$) ranging from 1.75 to 3.39, depending on the operating conditions (Table 3) where K_r_ is mass transfer coefficient of array of rough zinc discs; cm/s, and Ks is mass transfer coefficient of array of smooth zinc discs; cm/s. Both Fig. 10 and Table 3 demonstrate that once the surface roughness exceeds a peak-to-valley height of approximate 2mm, the effect of surface roughness reaches a limiting value with little increase in K with further increase in peak-to-valley height. The enhancing effect of surface roughness can be attributed to three coupled hydrodynamic and mass-transfer mechanisms that operate simultaneously under oscillatory conditions. (I) First, micro-eddy formation and localized hydrodynamic disturbances arise as the oscillatory radial flow interacts with the peaks and valleys of the rough surface. Flow separation at these asperities generates persistent micro-eddies that repeatedly disrupt the diffusion boundary layer, thereby thinning it and increasing the rate at which copper ions are renewed at the disc surface^49^. (II) Second, the enlarged geometric surface area associated with greater peak-to-valley height provides additional active sites for copper deposition. This expanded interfacial area improves the effective mass-transfer capacity of the disc and complements the hydrodynamic enhancement driven by eddy formation^49^. (III) Third, oscillatory flow induces periodic stretching, compression, and destabilization of the concentration boundary layer ($\delta$ )as it interacts with the surface asperities. This prevents the development of a stable diffusion layer and maintains a continuously refreshed interface, consistent with observations in oscillatory baffled and mechanically vibrated systems where structured surfaces significantly enhance convective mixing and mass-transfer efficiency^50^. Together, the synergistic contribution of increased surface area, continuous boundary-layer disruption, and micro-scale mixing provides a robust mechanistic basis for the superior mass-transfer performance of rough zinc discs compared with smooth ones. The emergence of a plateau beyond a roughness height of approximately 2 mm suggests that, beyond this scale, additional geometric modification does not generate further hydrodynamic or diffusive advantages under the applied oscillatory conditions.

**Table 3:** The enhancement ratio of array of rough zinc discs at different peak to valley height CuSO_4_ Conc. = 0.01 M and different vibration intensity.

V_i_, cm/s	Enhancement ratio (K_rough_/K_Smooth_)			
	P = 1 mm	P = 1.5 mm	P = 2 mm	P = 2.5 mm
15.83	2.08	2.38	3.31	3.39
31.67	1.85	1.95	2.38	2.44
47.12	1.8	2.25	2.82	3.12
62.99	2.06	2.62	3.06	3.16
78.79	1.84	2.34	2.74	2.96
94.25	1.75	1.96	2.21	2.44

The increase in the rate of cementation with increasing peak to valley in the case of presence of array of rough zinc discs indicates that array of rough zinc discs plays an important role in enhancing the rate of diffusion-controlled cementation.

### Effect of solution temperature

**Fig. 11:**
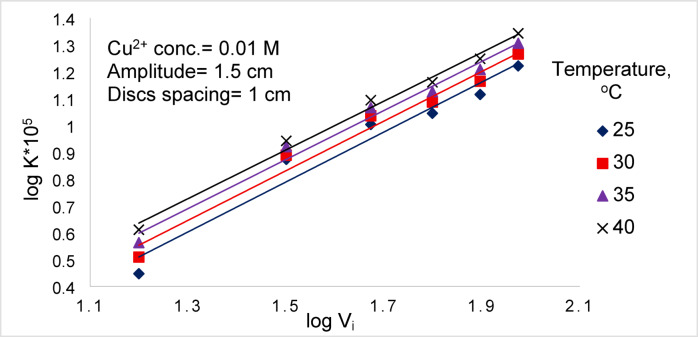
Effect of temperature at different vibration intensity.

Figure 11 shows that the cementation rate probably increases with increasing temperature. This trend can be attributed to the reduction in solution viscosity at elevated temperatures, which enhances ionic diffusivity in accordance with the Stokes–Einstein relation ($\frac{\mu D}{T}=const$)^12^. The resulting increase in diffusivity increases the mass-transfer coefficient ($K=\frac{D}{\delta }$)^12,33^, thereby increasing the rate of cementation.

**Fig. 12:**
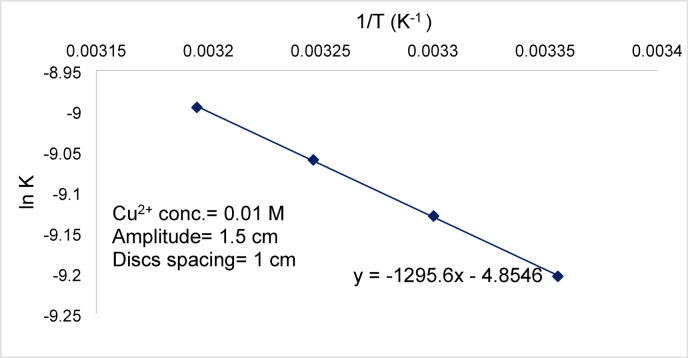
$$\mathrm{ln}K$$ vs. $$1/T$$

Figure 12 shows that the effect of temperature on the rate of cementation fits Arrhenius equation:

3 $$K={A}_{0}{e}^{-E/RT}$$

with an activation energy of 2.58 kcal/mole. This value confirms the diffusion-controlled mechanism of cementation of copper on zinc^12^.

### Mass transfer data correlation

In view of the turbulence nature of the present hydrodynamic conditions, dimensional analysis was used to correlate the present data. The mass transfer coefficient can be related to the different parameters for array of oscillating smooth zinc discs and array of oscillating corrugated zinc discs respectively according to the following equations:

4 $${\mathrm{K}} = f\left( { \, \rho ,\mu ,{\mathrm{D}},{\mathrm{d}}_{{\mathrm{c}}} ,{\mathrm{V}}_{{\mathrm{i}}} ,{\mathrm{S}}} \right)$$

5 $${\mathrm{K}} = f\left( {\rho ,\mu ,{\mathrm{D}},{\mathrm{d}}_{{\mathrm{c}}} ,{\mathrm{V}}_{{\mathrm{i}}} ,{\mathrm{S}},{\mathrm{P}}} \right)$$

By using dimensional analysis, the above equations can be written in terms of Sh, Re, Sc, S/d_c_,

S /d_c_ and P /d_c_ dimensionless group as follows

6 $${\mathrm{Sh}} = {\mathrm{a}}_{{\mathrm{o}}} {\mathrm{Re}}^{{\alpha {\mathrm{o}}}} {\mathrm{Sc}}^{\beta } \left( {{\mathrm{S}}/{\mathrm{d}}_{{\mathrm{c}}} } \right)^{{\gamma {\mathrm{o}}}}$$

7 $${\mathrm{Sh}} = {\mathrm{aRe}}^{\alpha } {\mathrm{Sc}}^{\beta } \left( {{\mathrm{S}}/{\mathrm{d}}_{{\mathrm{c}}} } \right)^{\gamma } \left( {{\mathrm{P}}/{\mathrm{d}}_{{\mathrm{c}}} } \right)^{\sigma }$$

where a_o_, a, α_o_, α, γ_o_, γ and σ are constants, which are determined from the present mass transfer data. The value of β was fixed at 0.33 according to previous studies un heat and mass transfer^12,25^.

**Fig. 13:**
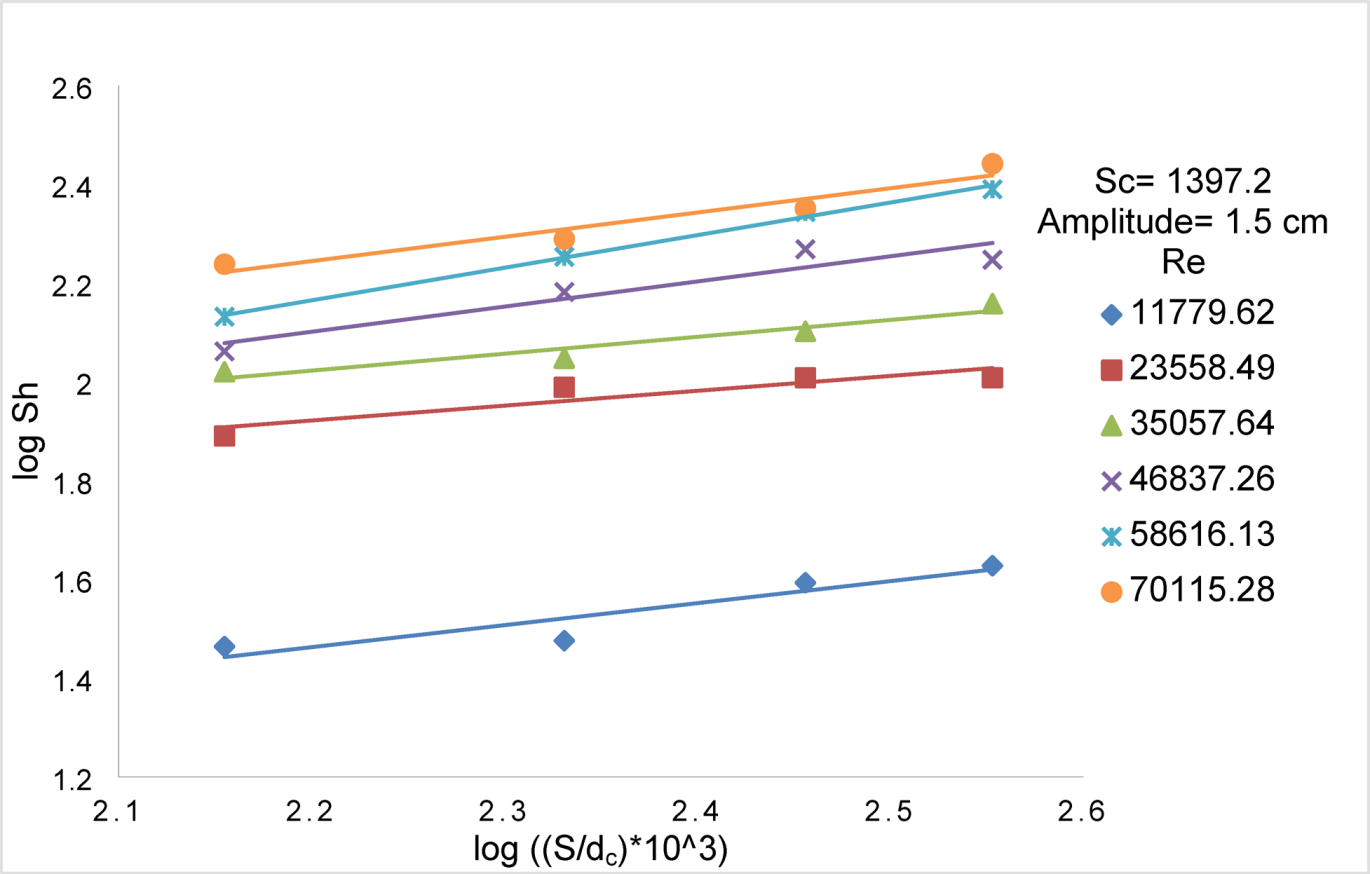
log Sh vs. log ((S/d_c_) × 10^3^) at different Re for array of smooth zinc discs.

**Fig. 14:**
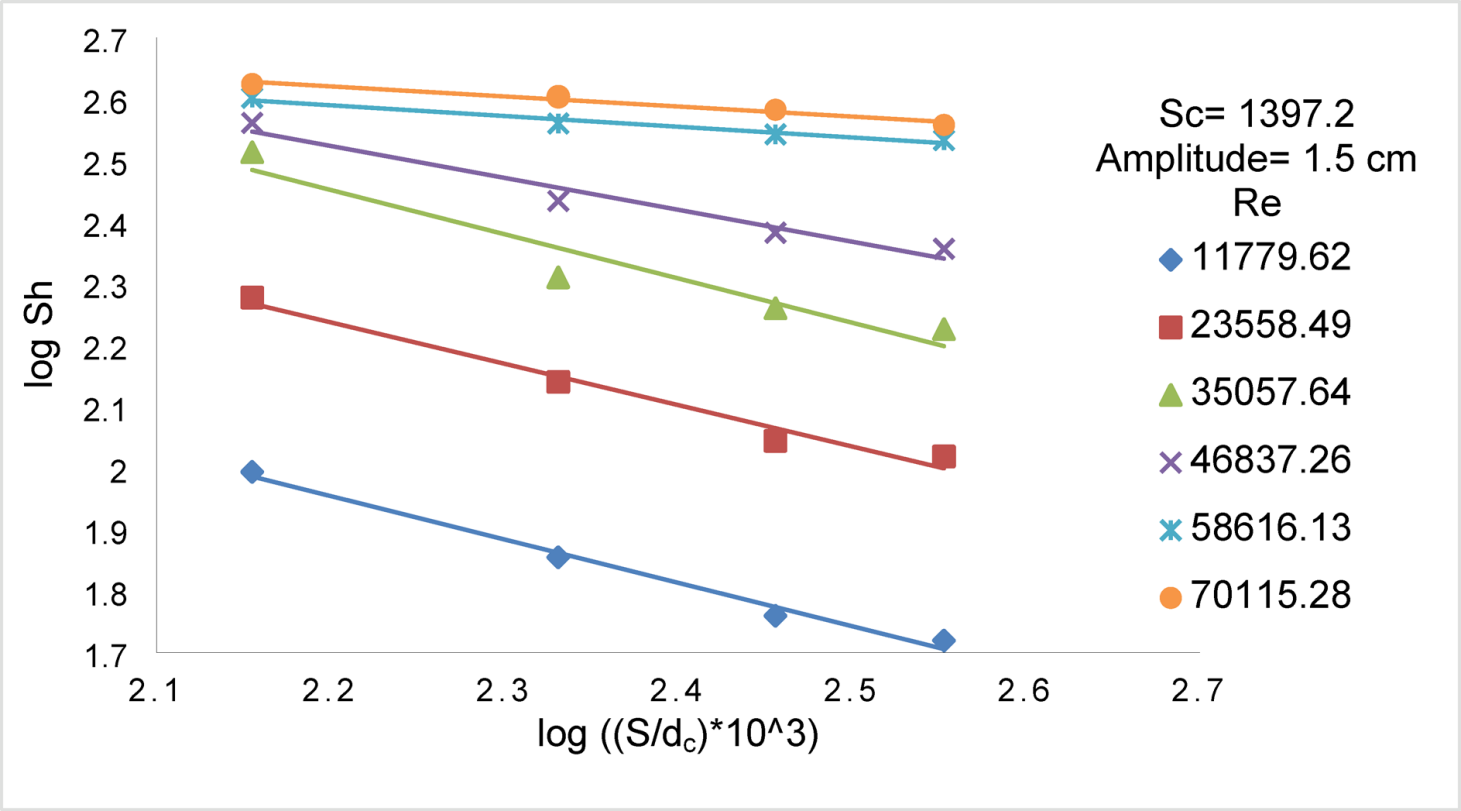
log Sh vs. log ((S/d_c_) × 10^3^) at different Re for array of rough zinc discs with peak to valley 2.5 mm.

**Fig. 15:**
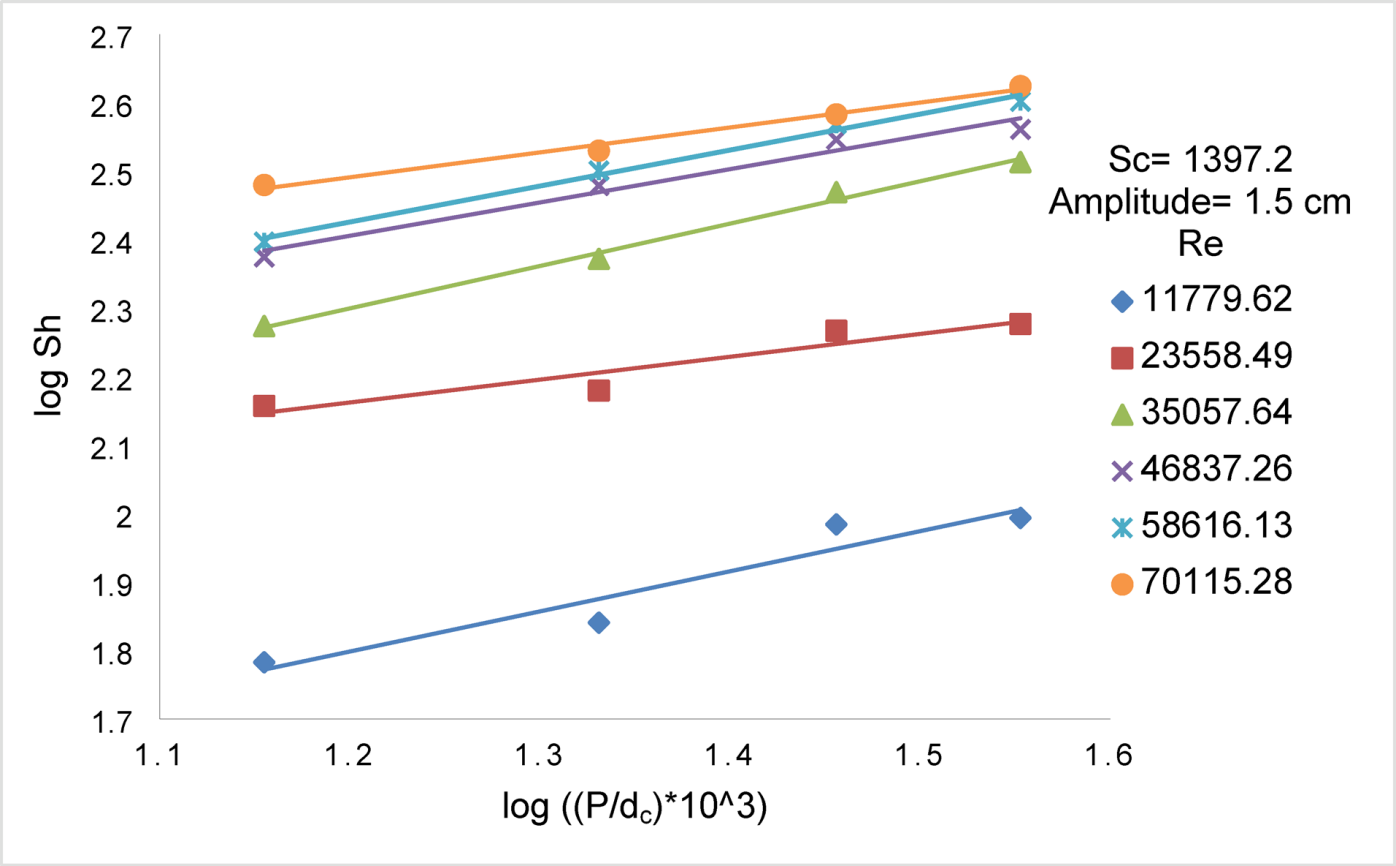
log Sh vs. log ((P/d_c_) × 10^3^) at different Re for array of rough zinc discs.

Figures 14 and 15 show the effect of Re on Sh for array of oscillating smooth zinc discs and array of oscillating rough zinc discs respectively. The Sh can be related to Re according to the following equations

8 $${\text{Smooth disc}}:{\mathrm{Sh}}\alpha {\mathrm{Re}}^{{{1}.0{6}}}$$

9 $${\mathrm{Rough}}\;{\mathrm{disc}}:{\mathrm{Sh}}\;\alpha \;{\mathrm{Re}}^{{0.{96}}}$$

whereas Fig. 13 shows the effect of (S / d_c_) on Sh in the case of using array of oscillating smooth zinc discs. The Sh can be related to (S/ d_c_) according to the following equation

10 $${\mathrm{Sh}}\alpha \left( {{\mathrm{S}}/{\mathrm{d}}_{{\mathrm{c}}} } \right)^{{0.{46}}}$$

whereas Fig. 14 shows the effect of (S / d_c_) on Sh in the case of using array of oscillating rough zinc discs. The Sh can be related to (S / d_c_) according to the following equation

11 $${\mathrm{Sh}}\alpha \left( {{\mathrm{S}}/{\mathrm{d}}_{{\mathrm{c}}} } \right)^{{ - 0.{49}}}$$

whereas Fig. 15 shows that Sh can be related to (P /d_c_) in the case of using array of oscillating rough zinc discs according to the following equation

12 $${\mathrm{Sh}}\alpha \left( {{\mathrm{P}}/{\mathrm{d}}_{{\mathrm{c}}} } \right)^{{0.{49}}}$$

**Fig. 16:**
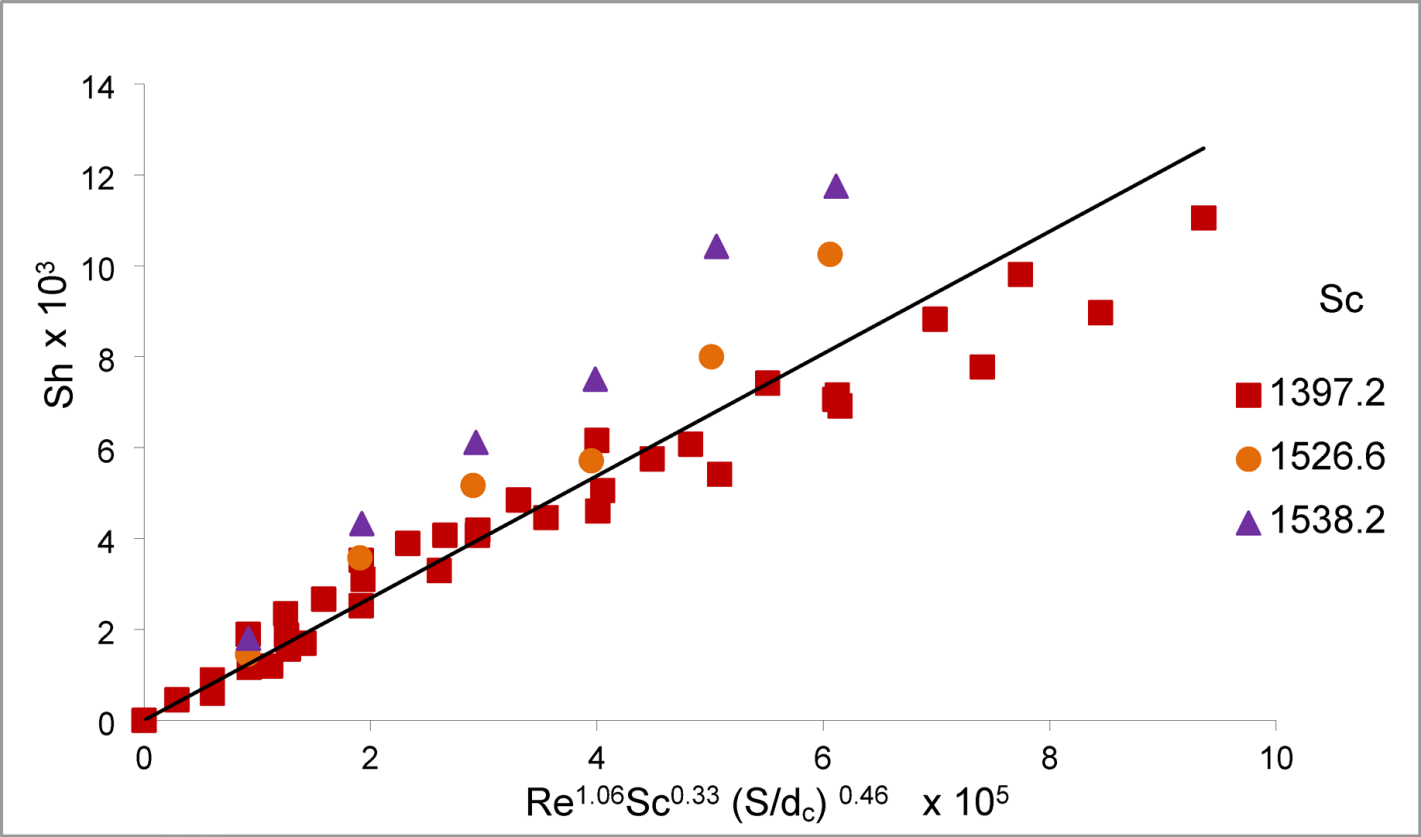
Overall mass transfer correlation for the cementation of copper on array of oscillating smooth Zinc discs.

Figure 16 shows that the present data for the conditions 3926.54 < Re < 70,115.28, 1397.2 < Sc < 1538.2, and 0.14 < S/d_c_ < 0.36 for array of oscillating smooth zinc discs fit the equation:

13 $${\mathrm{Sh}} = 0.0{\text{134 Re}}^{{{1}.0{6}}} {\mathrm{Sc}}^{{0.{33}}} \left( {{\mathrm{S}}/{\mathrm{d}}_{{\mathrm{c}}} } \right)^{{0.{46}}}$$

**Fig. 17:**
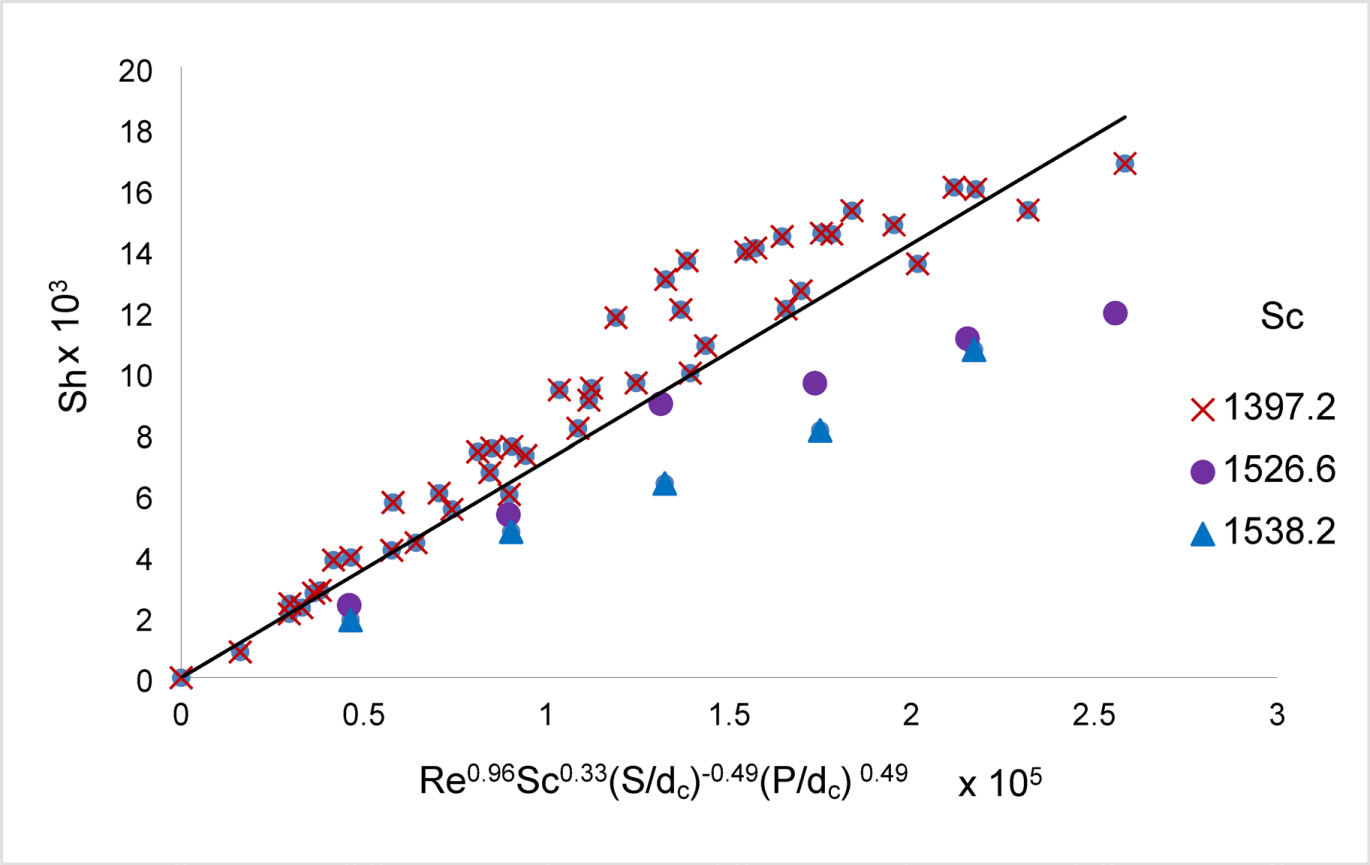
Overall mass transfer correlation for the cementation of copper on array of oscillating rough Zinc discs.

With an average deviation of ± 17.3%. Also, Eq. (13) can also be written in the form of:

Figure 17 shows that the present data for the conditions 3926.54 < Re < 70,115.28, 1397.2 < Sc < 1538.2, 0.14 < S/ d_c_ < 0.36, and 0.014 < P/ d_c_ < 0.036 for array of oscillating rough zinc discs fit the equation:

14 $${\mathrm{Sh}} = 0.0{\mathrm{711Re}}^{{0.{96}}} {\mathrm{Sc}}^{{0.{33}}} \left( {{\mathrm{S}}/{\mathrm{d}}_{{\mathrm{c}}} } \right)^{{ - 0.{49}}} \left( {{\mathrm{P}}/{\mathrm{d}}_{{\mathrm{c}}} } \right)^{{0.{49}}}$$

With an average deviation of ± 18%.

The present Re exponent 1.06 reveals a highly turbulent flow induced by pulsating solid discs. Perforated pulsating discs used by other authors gave Re exponents values of 0.537^12^ and 0.45^25^.

### Energetic considerations

It would be of interest to throw some light on the nature of turbulence that promotes the rate of mass transfer in the present case involving the equation derived by Calderbank and Moo Young based on the theory of entropy dissipating isotropic turbulence^51^. The authors correlated the mass transfer coefficient to specific energy dissipation (${\boldsymbol{\epsilon}})$by the equation.

15 $$K=0.13 {Sc}^{-0.66} {(\upnu \upepsilon )}^{0.25}$$

The equation is valid for turbulent flow mass transfer regardless of the geometry of the transfer surface or the method of turbulence generation.

Tojo et al.^40^ found that the power consumed in vibrating the horizontal disc is approximately by the equation.

16 $${P}_{i}\alpha {V}_{i}^{3}$$

The present study revealed that:

17 $$K \alpha {V}_{i}^{1.06}$$

From Eq. (16) it follows that ${\boldsymbol{\epsilon}}$ is given by:

18 $$\epsilon =\frac{{P}_{i} (watt)}{mass\, of\, the\, solution\, in \,the\, reactor \,(kg)}$$

i.e.

19 $$\epsilon \alpha {V}_{i}^{3}$$

Eliminating ${V}_{i}$ between Eqs. (17), (19) given:

20 $$K \alpha {\epsilon }^{0.32}$$

Comparing ${\boldsymbol{\epsilon}}$ exponents in Eqs. (20), (15) reveals that turbulence generated in the present study is not isotropic, it seems that large scale eddies are dominating the flow field, other turbulent flow mass transfer cases were found to deviate from Eq. (14).^52^ It is also probable that isotropic turbulence through which energy dissipation into heat take place is not uniformly distributed inside the reactor.

### Comparison of the present work with previous studies on cementation using different zinc geometries

**Fig. 18:**
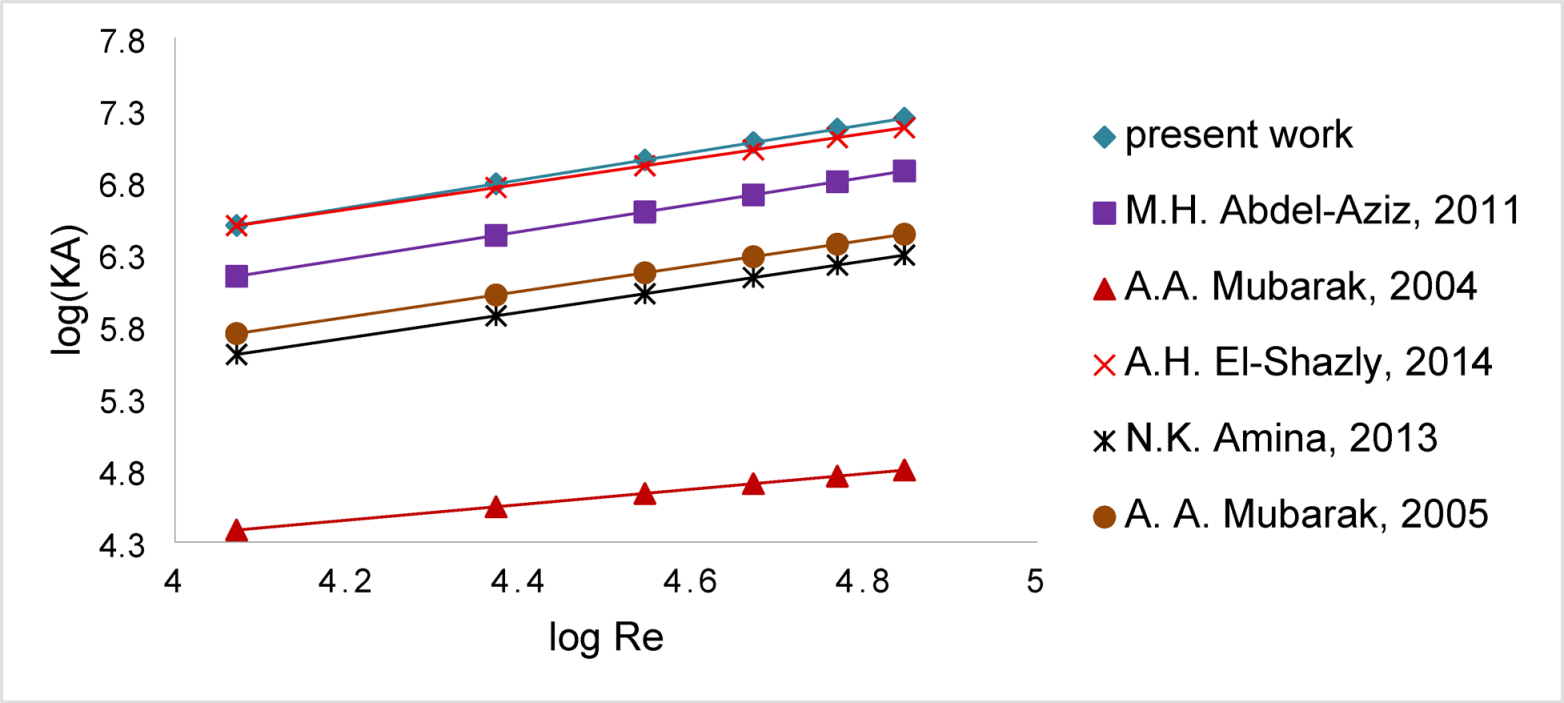
Comparison of Volumetric Mass Transfer Coefficient (kA) Between the Present Work and Previous Zinc Geometries in Cementation.

The volumetric mass transfer coefficient (kA) was used to compare the present work with those reported in previous studies employing zinc of different geometric shapes in the cementation process. As shown in Fig. 18, the use of an array of corrugated (rough) zinc discs in the present work resulted in KA values significantly higher than those reported by Mubarak et al.^12^ who used reciprocating horizontal perforated zinc discs, Amine et al.^9^ who used stationary zinc disc in an agitated vessel, Mubarak^53^ who used zinc sheet fixed to the wall of a batch agitated vessel, and Abdel-Aziz^19^ who used zinc Raschig rings packed in a rotating plexiglass perforated basket.

The current study slightly outperformed El-Shazly et al.^13^, who employed a vertical array of reciprocating perforated zinc discs with a total surface area approximately twice that of the present configuration. This indicates that the enhanced KA is not solely dependent on surface area but is strongly influenced by the geometric configuration of the zinc discs, which promotes improved turbulence, localized eddies, and higher surface renewal rates^49^. Furthermore, the corrugated and rough nature of the discs likely reduces the boundary layer thickness and enhances diffusion at the solid–liquid interface^50^, contributing to the observed increase in mass transfer efficiency.

### Sustainability and environmental implications

The present study demonstrates that copper ions can be effectively removed from industrial wastewater using zinc discs, with simultaneous reduction to metallic copper. The recovered copper can be re-melted, re-shaped, and reintegrated into a wide range of industrial applications, including electrical wiring, electronics, plumbing, and alloy manufacturing^1^, highlighting both its economic value and industrial relevance.

The vibration-assisted cementation process, combined with the use of rough zinc surfaces, significantly enhanced mass transfer rates and accelerated the copper removal process, leading to highly treated water and improved energy efficiency during operation.

In addition, dissolved zinc ions remaining in the water can also be recovered using established methods reported by Swee Su Lim et al.^54^ and Luchcińska et al.^55^, enabling the production of metallic zinc for reuse in water treatment or other industrial applications. Integrating these recovery strategies not only reduces the environmental impact of heavy metal discharge, but also promotes resource sustainability and supports the circular economy in industrial wastewater management.

## Conclusion

The present study revealed the following conclusions.

1. The results obtained show that vibratory agitation is powerful tool for increasing the rate of diffusion-controlled cementation. Copper ions could be effectively removed from synthetic waste water stream by cementation using array of oscillating rough zinc discs compared to array of oscillating smooth zinc discs. The mass transfer coefficient was found to depend mainly on concentration of copper ions, vibration intensity, spacing between zinc discs and peak to valley height of the rough disc. The rate of mass transfer and the rate of cementation were found to increase with increasing disc spacing and peak to valley height of the rough discs.
2. Disc array oscillation enhances the rate of mass transfer and the rate of cementation by a factor ranging from 5.8 to 61.4 compared to un-vibrated disc array depending on the operating conditions. Surface roughness cementation at rough oscillating array by a factor ranging from 1.75 to 3.39 compared to the smooth oscillating disc array.

The present dimensionless equation can be used to design and scale up the present bench scale reactor to an industrial scale reactor. The present reactor offers the advantages over the rotary reactors that it is more energy efficient^28,40^, compact (high area/ unit volume) and can be extended vertically to increase its capacity without extra floor space, this leads to decreasing the capital costs of the process.

## Data Availability

The datasets generated and analyzed during the current study are available from corresponding author on responsable request.

## List of symbols

A: Active area for array of zinc discs (cm^2^)
A_o_: Arrhenius constant
a_i_: Amplitude (cm)
C: Concentration at any time (M)
C_o_: Initial concentration (M)
D: Diffusion coefficient (cm^2^/s)
d_c_: Diameter of zinc disc (cm)
E: Activation energy (Kcal/mol)
f_i_: Frequency (RPS)
K: Mass transfer coefficient at the oscillating surface (cm/s)
K_o_: Mass transfer coefficient without vibration (cm/s)
K_r_: Mass transfer coefficient of array of rough zinc discs (cm/s)
K_s_: Mass transfer coefficient of array of smooth zinc discs (cm/s)
P: Peak to valley height of the rough surface (cm)
$${P}_{i}$$: Power (w)
Q: Solution volume (cm^3^)
S: Spacing between zinc discs in the array (cm)
T: Temperature (K)
t: Time (s)
V_i_: Vibrating intensity (cm/s)
R: Gas constant (atm l/mol k)
Re: Reynolds number ($\uprho {\mathrm{V}}_{{\mathrm{i}}} {\mathrm{d}}_{{\mathrm{c}}} /\upmu )$
Sc: Schmidt number (μ/ρD)
Sh: Sherwood number (Kd_c_/D)
$\mu$: Solution viscosity (g/cm s)
$\rho$: Solution density (g/cm^3^)
$\upepsilon$: Specific energy dissipation (w/kg)
Δ: Diffusion layer thickness (cm)
αo, α, β, γo, γ and σ: Constants
